# Entomophthovirus: an insect-derived iflavirus that infects a behavior-manipulating fungal pathogen of dipterans

**DOI:** 10.1093/g3journal/jkae198

**Published:** 2024-08-19

**Authors:** Maxwell C Coyle, Carolyn N Elya, Michael J Bronski, Michael B Eisen

**Affiliations:** Department of Molecular and Cell Biology, University of California Berkeley, Berkeley, CA 94720, USA; Department of Molecular and Cell Biology, University of California Berkeley, Berkeley, CA 94720, USA; Department of Molecular and Cell Biology, University of California Berkeley, Berkeley, CA 94720, USA; Department of Molecular and Cell Biology, University of California Berkeley, Berkeley, CA 94720, USA; Howard Hughes Medical Institute, University of California Berkeley, Berkeley, CA 94720, USA; Department of Integrative Biology, University of California Berkeley, Berkeley, CA 94720, USA

**Keywords:** entomopathogens, iflavirus, picornavirus, *Entomophthora*, metagenomics, behavior manipulation

## Abstract

We report a virus infecting *Entomophthora muscae*, a behavior-manipulating fungal pathogen of dipterans. The virus, which we name Berkeley Entomophthovirus, is a positive-strand RNA virus in the iflaviridae family of capsid-forming viruses, which are mostly known to infect insects. The viral RNA is expressed at high levels in fungal cells *in vitro* and during *in vivo* infections of *Drosophila melanogaster*, and virus particles can be seen intracellularly in *E. muscae*. This virus, of which we find two closely related variants in our culture of *E. muscae*, is also closely related to three different viruses reported from metagenomic surveys, two of which were isolated from wild dipterans, and a third isolated from wild ticks. By analyzing sequencing data from these earlier reports, we find abundant reads aligning to *E. muscae* specifically in the samples from which viral reads were sequenced. These data establish a wide and perhaps obligate association with *E. muscae* in the wild, consistent with our laboratory data that *E. muscae* is the host for these closely related viruses. Because of this, we propose the name Entomophthovirus (EV) for this group of highly related virus variants. As other members of the iflaviridae have been reported to cause behavioral changes in insects, we speculate on the possibility that EV plays a role in the behavioral manipulation of flies infected with *E. muscae*.

## Teaser

In this manuscript, Coyle *et al*. report the discovery of Entomophthovirus, a virus infecting the fungal pathogen *Entomophthora muscae. E*. muscae itself infects flies, notably manipulating their behavior in various ways during the course of infection. The authors show that this virus infects *E. muscae* in laboratory cultures, providing evidence from RNA sequencing, electron microscopy, and patterns of RNA fragments generated by the fungal immune system to combat viral infection. Furthermore, they show that Entomophthovirus co-occurs with previously undetected *E. muscae* infection in published metagenomic studies. This study opens new questions about *E. muscae* infection and evolution.

## Introduction

A wide variety of microbes have evolved the ability to manipulate animal behavior in ways that advance microbial fitness (Libersat 2003; Roy *et al.* 2006; Hoover *et al.* 2011; Forsythe *et al.* 2012; Rohrscheib and Brownlie 2013; Wang *et al.* 2015; Sampson and Mazmanian 2015; Eisthen and Theis 2016; Hughes *et al.* 2016). Among them is the fungal pathogen *Entomophthora muscae*. Originally identified in the 19th century (Cohn 1855), *E. muscae* has been observed infecting a wide variety of fly species (Kramer and Steinkraus 1981; Steinkraus and Kramer 1987), which exhibit a distinct series of behaviors prior to death: they climb to a high location (summiting), extend their proboscides to attach themselves to a substrate, and extend their wings in a characteristic “death pose,” allowing infectious conidia to shoot out and away from what is now a fly cadaver (Elya and De Fine Licht 2021).

A strain of *E. muscae* has been isolated from wild *Drosophila* and propagated in lab-reared *Drosophila melanogaster* and as an *in vitro* culture (Elya *et al.* 2018). *Drosophila melanogaster* infected by this strain of *E. muscae* manifest the same set of behavioral changes as have been described in other flies. Establishing an infection in this robust laboratory model has enabled the study of *E. muscae* infection by modern molecular genetic tools. Genetic screens have demonstrated elements of *Drosophila* neurocircuitry essential for the *E. muscae-*triggered behavioral response. These include specific neuronal populations, such as DN1p circadian neurons and pars intercerebralis to corpora allata projecting neurons, as well as the synthesis of juvenile hormone (Elya *et al.* 2023). Permeabilization of the blood–brain barrier is also likely important, as hemolymph from summiting flies can induce a burst of locomotion—a defining characteristic of summiting behavior—when isolated and transferred to uninfected flies (Elya *et al.* 2023). Yet much remains to be understood regarding the molecular identities and mechanisms of fungal virulence factors.

Here, we report that RNA sequencing of an in vitro *E. muscae* culture identifies a virus of the Iflaviridae family, part of the Picornavirales order of RNA viruses. The virus is absent from the *E. muscae* genome, consistent with Iflaviridae as RNA viruses and arguing against this virus being a domesticated endogenous element. This virus shows high similarity to viruses already reported in the literature, including Twyford virus (GenBank: KP714075.1), which was isolated during a survey of viruses in wild-caught *D. melanogaster* (Webster *et al.* 2015). Of the viruses isolated in that study, Twyford virus was unique in that the small RNAs derived from the virus showed a strong negative strand bias and a bias for a 5′ U base, uncharacteristic for small RNAs generated by *D. melanogaster* innate immunity. The authors explored the hypothesis that the virus was infecting a eukaryotic commensal of *Drosophila* but rejected various candidates: chelicerates because they have not been reported to display this viral RNA pattern, nematodes because they could not be detected by PCR in Twyford-carrying *Drosophila*, and *Neurospora* fungus because, although that fungus does indeed produce host genome-derived small RNAs with exactly those characteristics (22 nt, 5′-U), these small RNAs have not been associated with viral infection of *Neurospora* (Lee *et al.* 2010).

By sequencing small RNAs from our *E. muscae* culture, we show that the virus we identified has a similar small RNA profile to that of Twyford virus. Furthermore, by analyzing sequencing data from Webster *et al.* (2015), we show that hundreds of reads uniquely aligning to *E. muscae* are found specifically in the Twyford sample, suggesting that the virus they identified is in fact infecting *E. muscae.* Using published data from three additional studies—surveys of wild viruses in China and Australia, and a study of wild-caught *E. muscae* in Denmark—we show that wherever *E. muscae* appears in the wild, so does a virus closely related to both the virus we sequenced and to Twyford virus, while the opposite also holds true: almost all known instances of highly related viruses occur in samples with evidence of *E. muscae* infection. We used electron microscopy to show that this virus forms structures of approximately the same size as other Iflaviridae and that viral particles are found infecting *E. muscae* cells.

We name the virus we sequenced Berkeley Entomophthovirus (BEV), which encompasses 2 variants (BEV-a and BEV-b), and propose the name Entomophthovirus (EV) for the group of highly similar viruses that includes BEV and Twyford virus. The name reflects the host of the virus and its novelty as the first iflavirus known to infect a fungal pathogen of insects, or even any fungus at all. We also discuss the possibility that this virus plays a role in behavior manipulation.

## Materials and methods

### Culturing *E. muscae*

A liquid culture of *E. muscae* was propagated in Grace's Insect Media supplemented with lactalbumin hydrolysate, yeastolate, L-glutamine, and sodium bicarbonate (CAT #11-605-094, Fisher Scientific) and 5% fetal bovine serum (CAT #16-000-044, Fisher Scientific) as described in Elya *et al.* (2018).

### RNA sequencing, transcriptome assembly, and detection of EV

RNA was extracted from log-phase *E. muscae* liquid culture with Trizol (CAT #15596026, Thermo Fisher Scientific) using the manufacturer's protocol and treated with Turbo DNase (CAT #AM2238, Thermo Scientific) per the manufacturer's protocol. An RNA sequencing library was prepared using an Illumina TruSeq RNA V2 kit (CAT #RS-122-2001, Illumina) with 500 ng of input RNA. The library was sequenced to generate 37.9 million 150 bp paired-end reads on an Illumina HiSeq 4000 at the QB3 Vincent J. Coates Genomic Sequencing Facility at UC Berkeley. Raw reads are available through the National Center for Biotechnology Information (NCBI) Sequence Read Archive (SRA) as part of PRJNA1139198.

For transcriptome assembly, the 150 bp reads were trimmed to 100 bp from the 3′ end and assembled using Trinity v. 2.14.0 with the -jaccard_clip and -trimmomatic flags (Grabherr *et al.* 2011). Using blastn to search the genome of Twyford virus (GenBank: KP714075.1) against our de novo *E. muscae* transcriptome assembly revealed a transcript, TRINITY_DN192_c0_g2_i1, of 8,888 bp which aligns with 92.3% identity over 8,415 bp to Twyford virus (Supplementary File 1). A second transcript, TRINITY_DN192_c0_g1_i1, was also found with 82.4% identity to Twyford virus across 8,440 bp (Supplementary File 1). Both transcripts encode a single complete open reading frame (ORF) of 2,901 amino acids, which by PfamScan analysis (Mistry *et al.* 2021) encodes all of the characteristic proteins of an iflavirus, specifically coat proteins, a helicase, a protease, and an RNA-dependent polymerase (Supplementary File 1). We identify these as two variants of a virus we name BEV, with TRINITY_DN192_c0_g2_i1 as BEV-a and TRINITY_DN192_c0_g1_i1 as BEV-b.

### Phylogenetic tree of BEV and related Iflaviridae

**Fig. 1.**
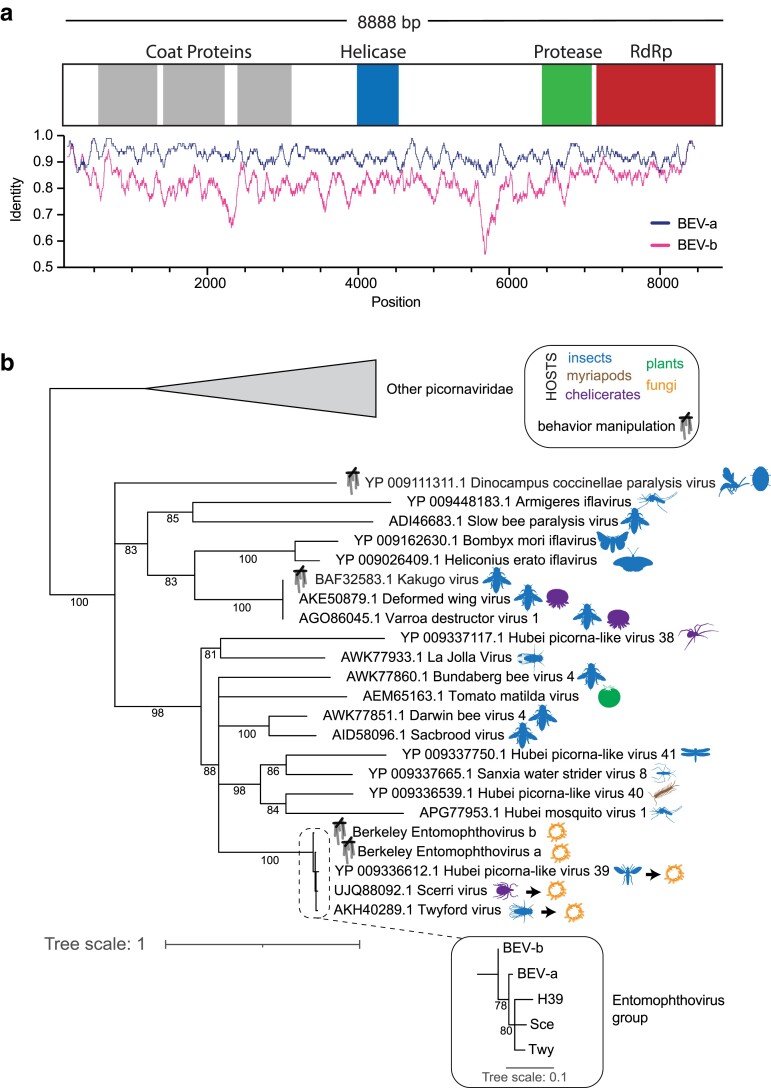
The genome and phylogenetic placement of EV. a) A schematic for the annotated genome of BEV, assembled by Trinity (v. 2.14.0) (Grabherr *et al.* 2011) from RNA sequencing of an in vitro *E. muscae* culture. The genome encodes a single polyprotein with the characteristic regions of an iflavirus, including three coat proteins, a helicase, a protease, and an RdRP (see Supplementary File 1 for genome sequence, ORF protein sequence, and domain annotation boundaries). Both BEV-a and BEV-b have an ORF of 2,901 amino acids with 90.6% identity and annotated protein domains in identical positions. Below the annotated viral domains is the similarity of each variant's genome to that of Twyford virus (Webster *et al.* 2015), using average pairwise identity across a 100 base pair sliding window. b) A maximum likelihood phylogeny of RdRP protein sequences clusters BEV (both variants) with Twyford virus, Scerri virus, and Hubei picorna-like virus 39 as a subclade of the Iflaviridae. Other characterized Iflaviridae, including some linked to behavior manipulation (marionette strings), are shown. Icons (from phylopic.org) specify the viral host (or close relative) as best known. For viruses identified by metagenomic shotgun sequencing [e.g. the Hubei and Sanxia viruses identified by Shi *et al.* (2016)], the viral host has not been confirmed. Arrows indicate viruses closely related to BEV for which a new host (namely *E. muscae* or closely related entomopathogenic fungi) is proposed here. Inset shows zoomed-in relationships for the EV group. GenBank identifiers label each viral sequence. Sequences were aligned with MAFFT (Katoh and Standley 2013), trimmed with ClipKIT (Steenwyk *et al.* 2020), and maximum likelihood phylogeny built by IQ-TREE (Nguyen *et al.* 2015). Support values were determined by 1,000 iterations of UltraFast bootstraps (Minh *et al.* 2013). Bipartitions with < 75% support are collapsed into palintomies. Taxon icons were downloaded from phloypic.org as black .svg files with attribution to the following authors and modifications to illustrations noted where required by license: Cyril Matthey-Doret (*Lysiphlebus fabarum* - CC0 1.0 Universal Public Domain Dedication), Melissa Broussard (*Coccinella undecimpunctata* - Attribution 3.0 Unported), T. Michael Keesey (*Culex* - Public Domain Mark 1.0), Lubna Maherally (*Apis mellifera*, *Varroa destructor* - CC0 1.0 Universal Public Domain Dedication), Gemma Martínez-Redondo (*Bombyx mori* - CC0 1.0 Universal Public Domain Dedication), Andy Wilson (*Agraulis vanillae*, *Libellula pulchella*, *Drosophila melanogaster* - CC0 1.0 Universal Public Domain Dedication), Cathy (*Latrodectus hasselti* - Attribution-NonCommercial-ShareAlike 3.0 Unported), Nicolas Gompel (*Sophophora suzukii* - rotated 90 degrees - Attribution-NonCommercial-ShareAlike 3.0 Unported), Twitter Emoji on Icon Scout (*Solanum lycopersicum* - Attribution 4.0 International), Tommasso Cancellario (*Gerris lacustris* - CC0 1.0 Universal Public Domain Dedication), Birgit Lang (*Lithobius forficatus* - CC0 1.0 Universal Public Domain Dedication), Levi Simons (*Entomophthoromycetes* - CC0 1.0 Universal Public Domain Dedication), Prespa Research Group (*Dasypogon diadema* - CC0 1.0 Universal Public Domain Dedication), Henry Lydecker (*Ixodes holocyclus* - rotated 90 degrees - Attribution-NonCommercial-ShareAlike 3.0 Unported).

We used the coding region of the BEV RNA-dependent RNA polymerase (RdRP) sequences and blastp to find homologous RdRp sequences among various Iflaviridae and other picornavirus outgroups. These RdRP sequences were aligned with MAFFT (v. 7.312) (Katoh and Standley 2013) using default options, trimmed with ClipKIT (v. 1.3.0) (Steenwyk *et al.* 2020) using the default smart-gap trimming mode, and trees were built with IQ-TREE (v. 2.2.0-beta COVID-edition) (Nguyen *et al.* 2015) using ModelFinder (Kalyaanamoorthy *et al.* 2017) and 1000 Ultrafast Bootstraps (Minh *et al.* 2013). Trees were visualized with iTOL (Letunic and Bork 2021). For display in Fig. 1b, the genomes of BEV variants and Twyford were aligned using MUSCLE (v. 3.8.425) (Edgar 2004) with a maximum of 8 iterations, implemented in Geneious.

### Analysis of *E. muscae* infected *D. melanogaster* RNA-Seq

**Fig. 2.**
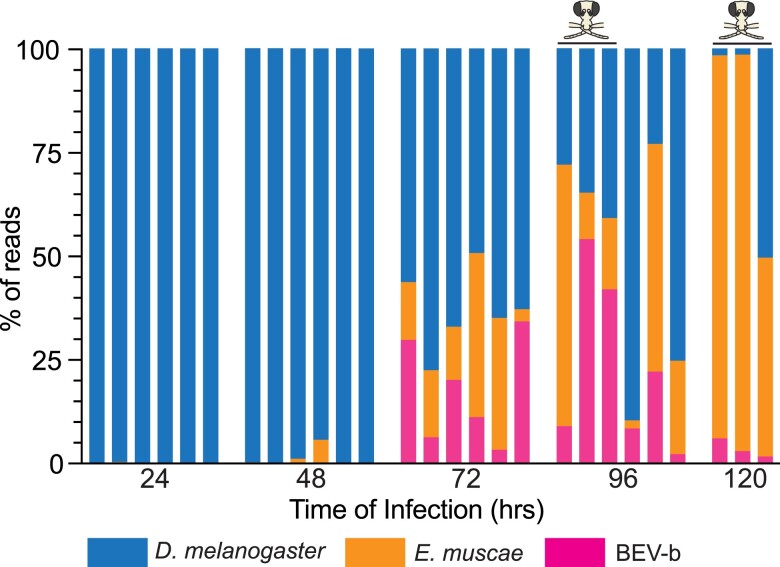
EV abundance in *E. muscae* infections of *D. melanogaster*. Using mRNA-Seq data from a time course of *E. muscae* infection in *D. melanogaster* (Elya *et al.* 2018), the fraction of reads in each sample aligning to *D. melanogaster*, *E. muscae*, and BEV-b genomes are shown. Samples were from individual whole flies exposed to *E. muscae* and sequenced at 24, 48, 72, 96, or 120 h after infection. Cadaver samples are marked with skull and crossbones. The proportion of reads aligning to BEV-b increases substantially at 72- and 96-h time points of the infection, concordant with the progression of *E. muscae* infection. However, infected fly cadavers at 120 h have a substantially lower viral burden. Reads aligning to BEV-a are not shown because they account for < 0.001% of reads in all samples (Supplementary File 2). All control flies had > 99.9% of reads aligning to *D. melanogaster*.

We used mRNA-Seq data from (Elya *et al.* 2018) available in the NCBI GEO database under ID GSE111046. Using bowtie2 (v. 2.5.4) (Langmead and Salzberg 2012), we created an index from a concatenated FASTA file combining (1) predicted transcripts from the most recent *E. muscae* genome assembly (Stajich *et al.* 2023), (2) predicted transcripts from the *D. melanogaster* r6.55 genome release accessed through FlyBase (Öztürk-Çolak *et al.* 2024), and (3) the genome sequences of BEV-a and BEV-b from our Trinity assembly. RNA-Seq reads were aligned to this combined index and read pairs aligning to each transcript were subsequently aggregated to get the total number of read pairs aligning to *E. muscae*, *D. melanogaster*, and the BEV variants. For each species, the read pairs aligning to that species were divided by the read pairs aligning to all species to get the proportions displayed in Fig. 2, which was generated with Prism software.

### Confirming virus in samples by RT-PCR

One milliliter of cultured cells was spun at 10,000*×g* for 5 min at room temperature to pellet fungal cells. The supernatant was filtered through a 0.22 µm syringe filter, and RNA was extracted with Trizol per the manufacturer's instructions. The pellet was resuspended in 10 mL phosphate-buffered saline (PBS) and vacuum filtered through a Whatman 0.8 µm cellulose ester filter (CAT # WHA10400912, Sigma Aldrich) to collect cells. This filter was washed 4 times with 10 mL of PBS. RNA was extracted from all washes with Trizol. The filter paper was equilibrated in 10 mL PBS for 30 min and the eluted cells were pelleted and RNA was extracted by Trizol. Additionally, Trizol extraction was performed on our media stocks and 25 CantonS flies from the fly stocks we have used to propagate *in vivo E. muscae* infections (Elya *et al.* 2018).

All RNA samples were reverse-transcribed with SuperScript III reverse transcriptase (CAT #18080093, Thermo Fisher Scientific) per the manufacturer's instructions using 150 ng of random hexamer primers per reaction. The RT reaction was heat-inactivated at 70°C for 15 min and one-tenth of the cDNA was used to amplify an 831-bp sequence specific to EV using Taq polymerase (CAT #M0273L, New England Biolabs) per manufacturer's recommendations. Amplification primers were, from 5′ to 3′, “GGGTTAGAAGTGTGCGAGAAT” and “GCGACAAGGACTACACGATAAG”. Amplicon presence was assayed with gel electrophoresis (1% agarose gel stained with ethidium bromide). Primers were originally designed using the Twyford virus genome and therefore do not align perfectly to either BEV genome as assembled by TRINITY. The cognate primers aligning perfectly to BEV-a are “GGATTAGAAGTGTGCGACAAT” (2 SNPs) and “GCGACATGGACTACATGATAAA” (3 SNPs). The corresponding primer binding sites in BEV-b have 6 mismatches each and therefore the amplification of BEV from the in vitro cultures is likely due to the detection of BEV-a specifically.

### Transmission electron microscopy

To prepare a crude sample of extracellular BEV, we pelleted 10 mL of *E. muscae* liquid culture, filtered the supernatant through a 0.22 µm syringe filter, and ultracentrifuged the sample for 2 h at 25,000 RPM and 4°C, using a SW 28 Ti swinging bucket rotor (Beckman Coulter). The pelleted material was fixed in 2.5% glutaraldehyde in 0.1 M sodium cacodylate, pH 7.4 (CAT #16537-20, Electron Microscopy Sciences) and a 1:100 dilution was negative-stained with 1% uranyl acetate (CAT #22400-1, Electron Microscopy Sciences) and imaged on a Tecnai 12 TEM (Field Electron and Ion Company).

To image sections of *E. muscae* cells, pelleted cells were fixed in 2.5% glutaraldehyde in 0.1 M sodium cacodylate, pH 7.4, briefly centrifuged and embedded in a drop of 2% agarose. Samples were washed with 0.1 M sodium cacodylate (CAT #C0250, Sigma Aldrich), then incubated with 1% osmium tetroxide (CAT #201030, Sigma Aldrich) and 1.6% potassium ferricyanide (CAT #702587, Sigma Aldrich) for 30 min then washed again with 0.1 M sodium cacodylate. Fixed and embedded samples were then dehydrated with increasing concentrations of ethanol, followed by pure ethanol and then pure acetone. Increasing concentrations of EPON resin (CAT #14120, Electron Microscopy Sciences) in acetone (25, 50, 75, then 100%) were infiltrated into the samples for 1 h each, followed by pure resin infiltration overnight. Then EPON resin with 2.5% benzyldimethylamine accelerant (CAT #11400, Electron Microscopy Sciences) was infiltrated into samples for 5 h. Samples were embedded into a mold and incubated at 60°C for 1 week. Seventy nanometer sections of samples were cut and stained with 2% uranyl acetate, followed by Reynolds lead citrate (CAT #22410-01, Electron Microscopy Sciences), before imaging on a Tecnai 12 TEM (Field Electron and Ion Company).

### Small RNA sequencing

Four milliliters of *E. muscae* liquid culture was pelleted and RNA was extracted with Trizol per manufacturer's instructions. The sample was treated with Turbo DNase and Trizol extracted again. RNA integrity was confirmed with an RNA 6000 Pico chip (CAT #50671513, Agilent Technologies) on the Agilent 2100 Bioanalyzer. RNA was diluted to 200 ng/µL, and 1 µg (5 µL) was used as input for the TruSeq small RNA kit (CAT #RS-200-0012, Illumina). The size range was confirmed on the 2100 Bioanalyzer with a HS DNA chip (CAT #50674626, Agilent Technologies). 204.5 million 50 bp unpaired reads were obtained with a HiSeq 4000 (Illumina).

Cutadapt software (Martin 2011) was used to trim 3′ bases with a Phred score ≤ 10 and to remove the 3′ Illumina small RNA adapter. Next, cutadapt was used to select sequences between 17 and 29 bp and these reads were aligned to both the BEV-a and BEV-b genomes with bowtie2 v.2.5.4 (Langmead and Salzberg 2012). Samtools (v. 1.20) (Danecek *et al.* 2021) was used to extract reads aligning to the forward or reverse strands of either viral variant, and data were pooled between the variants. Read lengths and 5′ base identities were aggregated using the Biopython package in Python (Cock *et al.* 2009). Prism software was used to plot the proportions of each small RNA category defined by 5′ base identity and length, combining our data for BEV with that from Webster *et al.* (2015) for Twyford virus and Kilifi virus.

### Analysis of studies reporting EV relatives

Three closely related viruses to BEV had previously been reported in the literature: Twyford virus (Webster *et al.* 2015) from a survey of wild-caught *Drosophila*, H39 from a broad survey of invertebrate viruses (Shi *et al.* 2016), and Scerri virus from *Haemophysalis bancrofti* ticks in Australia (Gofton *et al.* 2022). For each of these studies, we obtained the original reads from NCBI's SRA. For Twyford (NCBI BioProject PRJNA277921), we analyzed two samples: SRR1914527, the sample from which Twyford virus was identified, and SRR1914484, which was not reported to contain Twyford virus (Webster *et al.* 2015). For H39 (NCBI BioProject PRJNA318834), we analyzed all 67 samples from the viral survey (Shi *et al.* 2016). For Scerri (NCBI PRJNA777641), we analyzed all samples from *H. bancrofti* ticks, the only tick species in which Scerri virus was reported to be found (Gofton *et al.* 2022).

We aligned reads using bowtie2 (v. 2.5.4) (Langmead and Salzberg 2012) with default parameters to a set of all mRNA transcripts (*n* = 43,451) from a recently published assembly and annotation of the *E. muscae* genome (Stajich *et al.* 2023). Initial results showed that reads from all samples were aligning promiscuously to a small number of transcripts in the annotation. After manual inspection and blastn searches, we found that these corresponded to repeats (e.g. GAA, GAAA, polyA, and polyC) and highly conserved housekeeping genes, including actin, tubulin, histones, ATP synthase, 60S ribosomal genes, and mitochondrial electron-transport genes. Reads aligning to this set were filtered from all samples (Supplementary File 4 details each transcript for which aligning reads were filtered and why).

For each SRA sample, we also aligned reads to the respective published viral genome: Twyford (GenBank: KP714075.1), H39 (GenBank: KX883974.1), or Scerri (GenBank: OL452259.1). For plotting in Fig. 4d, we normalized the viral counts by the total reads in a given sample, multiplied by 1 million. We also added a pseudocount of 0.1 reads per sample for visualization on log scale. Plots were made in Prism software. Reads aligning to *E. muscae* were also normalized per million total reads. Full data shown in Supplementary File 4.

### Identification of *Delia radicum* and *Musca domestica* EV

A previous study generated RNA sequencing reads from confirmed *E. muscae* infections of wild-caught cabbage flies (*D. radicum*) and of laboratory infected house flies (*M. domestica*) (De Fine Licht *et al.* 2017). Two strains of *E. muscae—*KVL-14-117 and KVL-14-118—had been used to infect *M. domestica*, with fungal *in vitro* cultures further propagated for KVL-14-117. To look for EV infections in these samples, we accessed these reads (NCBI BioProject ID PRJEB10825) as well as five transcriptomes made available by the authors—one curated transcriptome each for KVL-14-117 and KVL-14-118, as well as a transcriptome for each of the three wild-caught *D. radicum* samples. A full-length virus aligning to BEV was identified in one of the *D. radicum* samples by blastn, which we call DrEV (‘see *Results*’). The authors of De Fine Licht *et al.* (2017) note that they had filtered the transcriptomes of KVL-14-117 and KVL-14-118, including removing transcripts most closely aligning to viral sequences, so we regenerated transcriptomes from the RNA sequencing reads using Trinity 2.14.0 with the -trimmomatic flag and -min_contig_length set to 150 (Grabherr *et al.* 2011). Using blastn, we identified partial (KVL-14-117) and full-length (KVL-14-118) EV hits in these transcriptomes, which we named MdEV-117, MdEV-118a, and MdEV-118b. To determine viral abundance in these samples, we used bowtie2 (v. 2.5.4) (Langmead and Salzberg 2012) with default parameters to align RNA sequencing reads to the respective viral genome: DrEV, MdEV-117, and MdEV-118a/MdEV-118b.

## Results

### Discovery of an iflavirus in in vitro culture of an isolate of *E. muscae*

We previously described the isolation of a strain of *E. muscae* from wild *Drosophila* caught in Berkeley, CA in the summer of 2015 (Elya *et al.* 2018). We had generated an *in vitro* culture by capturing and germinating spores ejected from an individual *D. melanogaster* recently killed by *E. muscae* (Hajek *et al.* 2012; Elya *et al.* 2018).

From this *in vitro E. muscae* culture, we sequenced mRNA, obtaining 38.9 million 150 bp paired-end reads. We first noticed that individual reads analyzed by blastn showed ∼ 95% identity to Twyford virus (GenBank: KP714075.1), which was identified in a survey of viruses of wild *Drosophila* (Webster *et al.* 2015). After assembling an *E. muscae in vitro* transcriptome using TRINITY (Grabherr *et al.* 2011), we used blastn to compare all of the assembled transcripts against Twyford and found two highly similar viral genomes with high identity to Twyford virus (Supplementary File 1). The first, which we refer to here as BEV-a, has 92.3% identity to Twyford virus across an 8,415 bp alignment (Fig. 1a). The second variant, BEV-b, has 82.6% identity to Twyford across 8,440 bp. Both BEV-a and BEV-b have a length of 8,888 bp and encode a single predicted ORF of 2,901 amino acids, which are 90.6% identical to one another (Supplementary File 1).

The single viral pro-protein encoded by these ORFs contains the six proteins characteristic of Iflaviridae: three coat proteins, an RNA helicase, a protease, and an RdRP (Fig. 1a, Supplementary File 1). We built a maximum likelihood phylogenetic tree to compare BEV to other Iflaviridae and Picornaviridae, using coding sequences of the RdRP (Fig. 1b, Supplementary File 1). The BEV variants cluster closely with Twyford and two other viruses: Scerri virus and Hubei picorna-like virus 39.

### BEV RNA increases in abundance during in vivo infections of *D. melanogaster* with *E. muscae*

Reads aligning to the BEV genome account for 11.8% of the reads obtained from the *in vitro* culture. BEV-a accounts for 7.6% of the reads while BEV-b accounts for 4.2%. This shows that the virus is capable of survival in fungal *in vitro* cultures, where it may be actively replicating or transmitted vertically across fungal cell divisions. We also analyzed a previously published dataset of RNA sequenced over the course of an *E. muscae* infection (Elya *et al.* 2018). In *D. melanogaster*, infections by *E. muscae* are first evident as an opaque abdomen beginning at 72 h, while most flies die at 96 or 120 h after exposure (flies typically die at sunset as defined by a 12:12 light:dark cycle; cadavers marked in Fig. 2a) (Elya *et al.* 2018). We found BEV-b (but not BEV-a) RNA in late infection stages of flies infected with *E. muscae* (Fig. 2, Supplementary File 2), tracking alongside an increase in the reads aligning to *E. muscae* itself. There is, however, considerable inter-animal variation: at 72 h between 3.3 and 34.3% of all reads align to BEV-b, and at 96 h the range is 9.0 to 54.2% for cadavers, while for living flies it is 2.2 to 22.2%. At 120 h, when all flies sampled were cadavers, the fraction of reads aligning to BEV drops while those aligning to *E. muscae* continues to rise. Uninfected controls had > 99.9% of reads aligning to *D. melanogaster* (Supplementary File 1).

### Evidence of intracellular and extracellular virus in *E. muscae* in vitro culture

**Fig. 3.**
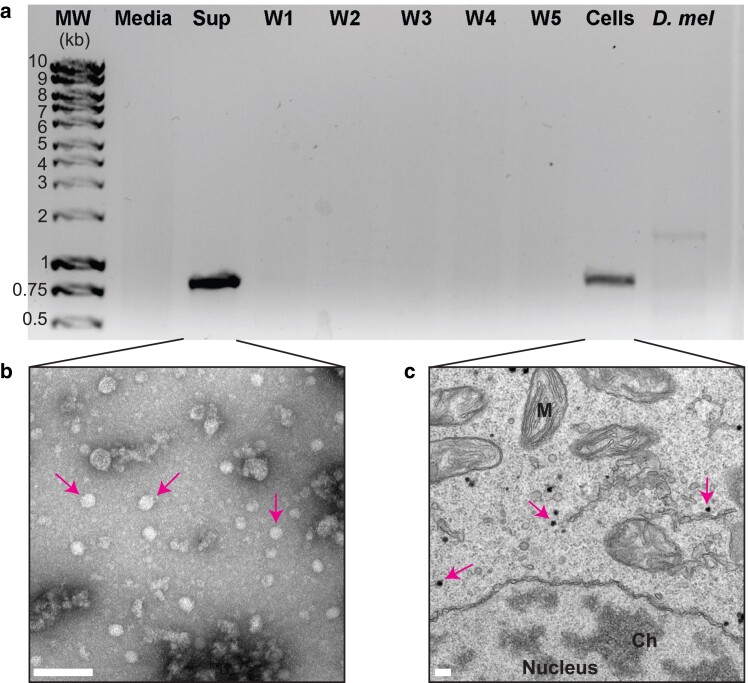
EV is present in *E. muscae* culture. a) RT-PCR with primers specific for the Twyord virus genome (‘see *Materials and Methods*’) show that BEV is in the supernatant (Sup) and cellular fraction (Cells) of an *in vitro* culture of *E. muscae*. Viral signal is lacking from the media used for *in vitro* culture (Media), the CantonS *Drosophila* stocks used to propagate the infection in lab (*D. mel*), and washes of the cellular fraction (W1–W5). The amplified viral fragment is 831 bp. b) TEM of virus collected from the supernatant of *i*n vitro** cultures by ultracentrifugation and negative-stained by uranyl acetate. The viral particles (arrows) have the expected diameter (∼ 30 nm) of an iflavirus capsid. Scale bar = 100 nm. c) TEM of cellular sections of *E. muscae*, with a double-contrast staining of uranyl acetate and lead citrate. Electron-dense particles (arrows) consistent with the size distribution of an iflavirus capsid are abundant in some cellular sections and are localized in the cytoplasm. The *E. muscae* nucleus, mitochondria (M), and chromatin (Ch) are also marked. Scale bar = 100 nm.

At this point our only evidence for the existence of BEV in *E. muscae* was through RNA sequencing, so we sought to visualize viral particles in the *in vitro* culture. We spun down fungal cells and filtered the supernatant through a 0.22 µm filter to retain potential viral particles. Using primers to specifically amplify an 831 bp segment of the BEV genome after reverse transcription, we found evidence for BEV in the supernatant (Fig. 3a). We then resuspended the cell pellet and collected cells on a filter by vacuum filtration. After thoroughly washing the cells by vacuum filtration, followed by recovering cells from the filter, we found a strong signal for BEV in the eluted cell fraction, but not in any of the washes (Fig. 3a). No viral signal was detected in the media used to culture *E. muscae* or in stocks of the CantonS flies that had been used for *in vivo E. muscae* infections. Together this demonstrates that the presence of BEV is specifically associated with *E. muscae* in both the culture supernatant and inside of cells.

**Fig. 4.**
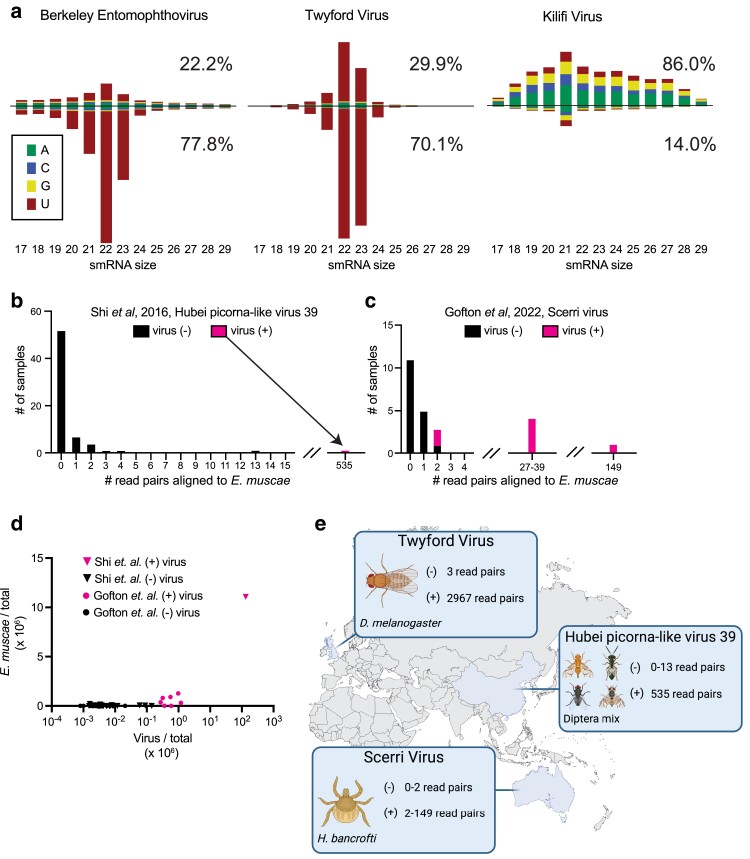
EV family members co-occur with *E. muscae* infection globally. a) Small RNAs generated from BEV in *E. muscae* show a nucleotide bias like that of Twyford virus and different from other viruses infecting *Drosophila*. Following the presentation of data from Webster *et al.* (2015), the length and 5′ nucleotide bias of aligned reads is shown, with the data from Webster *et al.* (2015) for Twyford virus and Kilifi virus shown alongside. Kilifi virus is a *Drosophila*-infecting picornavirus. For both BEV and Twyford, the aligned reads are mostly 21–23 nt, with a negative strand bias (shown numerically in figure) and a strong 5′ U bias. Data for generating graphs are in Supplementary File 3. b) A survey of diverse invertebrate viruses (Shi *et al.* 2016) identified a virus (Hubei picorna-like virus 39) closely related to BEV (see Fig. 1b) in a sample of wild dipterans from Hubei province, China. The reads from this sample also contain 535 read pairs aligning to the *E. muscae* genome (after filtering for repeats and highly conserved housekeeping genes, ‘see *Materials and Methods*’). Also shown are the number of read pairs aligning to *E. muscae* in the 66 other samples sequenced in this study. c) A survey of metatranscriptomes from Australian ticks (Gofton *et al.* 2022) identified a virus (Scerri Virus) closely related to BEV (see Fig. 1b) in samples of *H. bancrofti* ticks from Kioloa. Out of 24 samples of *H. bancrofti*, 7 contained multiple read pairs aligning to Scerri Virus. Shown are the number of read pairs aligning to *E. muscae* for the 17 viral-negative samples and the 7 viral-positive samples. d) The data from (b) and (c) are plotted, normalizing the abundance of reads for both virus and *E. muscae* by 1 million reads sequenced for each sample. For plotting on log scale, a pseudocount of 0.1 viral reads was added to all samples with 0 viral reads. e) A map showing the countries in which Entomopthovirus has been detected, as well as the reported host taxa and the range of read pairs aligning to *E. muscae* in viral-positive and viral-negative samples from each study.

We used TEM to visualize the presence of virus-like particles, first in a sample collected from the *in vitro* supernatant (Fig. 3b). Iflavirus capsids have been reported to have a diameter of around 30 nm (Silva *et al.* 2015), and the regular size and shape of viral particles help them stand out by TEM. Indeed, a uranyl acetate negative stain of viral particles collected by ultracentrifugation from the extracellular fraction showed an abundance of symmetric ∼30 nm objects, consistent with the capsids of an iflavirus (Fig. 3b).

We next carried out double-contrast uranyl acetate/lead citrate staining of fixed *E. muscae* cells to look for virus particles. Many sections had intracellular particles consistent with viral capsids by virtue of their ∼30 nm diameter and their strong electron density (Fig. 3c). These particles were never found inside of the nucleus, mitochondria, or other clearly demarcated organelles (Fig. 3c).

### *E. muscae* is present in the Twyford samples

Having established that BEV is present in *E. muscae* cells *in vitro* as well as during infections of *D. melanogaster*, we looked for evidence of *E. muscae* in those samples from which viruses closely related to BEV have been identified, specifically Twyford virus, Scerri virus, and Hubei picorna-like virus 39 (Fig. 1b). Beginning with Twyford virus, we obtained reads for the original samples from the NCBI's SRA: SRR1914527, which contains flies infected with Twyford virus and SRR1914484, which does not contain Twyford virus (Webster *et al.* 2015).

We aligned reads to annotated mRNAs from a recently published *E. muscae* genome (Stajich *et al.* 2023), filtering out alignments to repeat-rich sequences (polyA, polyC, GAA, and GAAA) and highly conserved housekeeping genes such as actin, tubulin, and histones (‘see *Materials and Methods*’; Supplementary File 4). We found 2,967 read pairs that align to *E. muscae* in the Twyford sample, hitting 2,018 distinct genes, while there are only 3 aligning reads in the non-Twyford sample (Supplementary File 4).

As described above, Webster *et al.* (2015) had noted an unusual profile of small RNAs isolated from the Twyford sample that aligned to the Twyford virus genome. We sequenced small RNAs from our *in vitro* culture and found that the small RNAs generated from BEV (combining data from both variants) have very similar properties to those described for Twyford (Fig. 4a, Supplementary File 3). They show a strong (>70%) negative strand bias with a preference for a 5′ U base. On the other hand, picornaviruses that infect *Drosophila* in the absence of *E. muscae* display a much different distribution of small RNAs, with a positive-strand bias and less specificity for length and 5′ base (e.g. Kilifi virus, Fig. 4a; Webster *et al.* 2015). Furthermore, the *E. muscae* genome contains clear homologs of Dicer (e.g. GenBank KAJ9053924.1, with 28% amino acid identity to *H. sapiens* Dicer and a BLAST *e*-value < 1*e*^−82^), suggesting that small RNAs may be processed by a Dicer pathway in *E. muscae*. Collectively this evidence demonstrates that Twyford virus and BEV represent variants of the same *E. muscae*-infecting virus. We propose naming this virus Entomophthovirus (EV).

### EV in additional surveys of invertebrate viruses

GenBank contains two other viruses closely related to BEV and Twyford: Hubei picorna-like virus 39 (H39; 81.9% identity to BEV-a; KX883974.1) and Scerri virus (88.6% identity to BEV-a; OL452259.1). H39 was identified as part of a large survey of viruses from different collections of invertebrate taxa in Hubei province, China (Shi *et al.* 2016). Scerri was identified in a meta-transcriptomic survey of Australian ticks (Gofton *et al.* 2022). For both studies, we obtained their raw sequencing reads and aligned them to the respective BEV-related virus and to the *E. muscae* transcriptome.

The sample from which H39 was identified, a mixture of unspecified wild dipterans, contains 6,408 read pairs that align to H39 as well as 535 read pairs that align to 418 different *E. muscae* transcripts (Fig. 4c and e, Supplementary File 4). Of the remaining 66 samples from Shi *et al.* (2016), 65 contained 0–4 read pairs aligning to *E. muscae*, while one sample contained 13 read pairs aligning to *E. muscae*. Three samples (out of 66) contained 2–4 read pairs aligning to H39, all from samples without *E. muscae*-aligning read pairs (Fig. 4b and d, Supplementary File 4). In short, the only sample with a strong signal for H39 also showed a strong signal for *E. muscae*.

For Scerri virus, 7 out of 24 *H. bancrofti* tick pools from Gofton *et al.* (2022) contained multiple read pairs aligning to the Scerri virus genome (Fig. 4c–e, Supplementary File 4), which is itself fragmented, consistent with the lower abundance of viral reads in these samples when compared to the samples that generated full-length H39 and Twyford virus assemblies. Of the seven viral-positive samples, five samples had similar numbers of read pairs (27–149) also aligning to *E. muscae*, hitting 16–79 different transcripts, while two viral-positive samples had only two read pairs aligning to *E. muscae* (Fig. 4c–e, Supplementary File 4). Of the 17 viral-negative samples, 11 samples had zero read pairs aligning to *E. muscae*, five samples had one aligning read pair, and one sample had two aligning read pairs (Fig. 4c–e, Supplementary File 4). Overall, the trend is that most viral-infected samples show evidence of *E. muscae* infection, while *E. muscae* reads are not found above background levels in uninfected samples.

Another recent study sequenced RNA from cabbage flies (*D. radicum*) and house flies (*M. domestica*) known to be infected with *E. muscae* (De Fine Licht *et al.* 2017). Three separate *E. muscae* transcriptomes were built in that study from individual *D. radicum* flies infected with *E. muscae.* All three transcriptomes have a transcript that aligns for over 3,900 bp with >80% identity to both BEV-a and BEV-b, including one full-length EV (87.2% identity with BEV-a over 8,827 bp and 83.0% identity with BEV-b over 8,837 bp; GenBank locus GENB01034640), which we henceforth refer to as DrEV (Supplementary File 4). The three *D. radicum* samples contained 102, 124, and 131,925 read pairs aligning to DrEV, respectively (Supplementary File 4).

For *M. domestica*, three flies were infected with one *E. muscae* strain (KVL-14-117), which was also used to seed three in vitro *E. muscae* cultures, while three other flies were infected with a distinct *E. muscae* strain [KVL-14-118; (De Fine Licht *et al.* 2017)]. We built de novo transcriptomes from RNA reads from infected flies for both KVL-14-117 and KVL-14-118, as the original transcriptomes from De Fine Licht *et al.* (2017) had intentionally filtered out transcripts most closely aligning to viruses. Our transcriptome from KVL-14-117 contains a truncated virus sequence (MdEV-117) of 3,966 bp aligning to both BEV-a variants with >81% identity across >3,900 bp (Supplementary File 4). Our transcriptome for KVL-14-118 contained 2 EV variants. MdEV-118a aligns most closely to BEV-a (87.7% across 8,839 bp) while MdEV-118b aligns most closely to BEV-b (86.8% identity across 8,829 bp; Supplementary File 4). The three samples infected with KVL-14-117 contained 107, 207, and 174 reads aligning to MdEV-117, while the 3 flies infected with KVL-14-118 contained 69,322/629,708, 135,014/1,234,496, and 67,434/141,867 reads aligning to MdEV-118a/MdEV-118b, respectively, representing a much higher viral load (Supplementary File 4). Intriguingly, the three *in vitro* cultures seeded from the lower burden MdEV-117 *in vivo* infections yielded zero reads aligning to MdEV-117, suggesting that the virus could be cleared in these samples (Supplementary File 4).

Finally, we searched for BEV and related viruses (Twyford, Scerri, and H39) in transcriptome data from flies at various stages of infection with a variety of different pathogenic bacteria to explore whether EV might be an opportunistic infection in flies undergoing immune collapse (Troha *et al.* 2018). We did not find any reads aligning to BEV-a, BEV-b or any other EV variant (Twyford, H39, and Scerri), consistent with the observation from Webster *et al.* (2015) that Twyford virus is rare in *Drosophila*.

In summary, our survey of currently published sequencing data suggests that EV is obligately associated with *E. muscae* in *in vivo* infections. We cannot find evidence for EV infection where *E. muscae* infection was not confirmed phenotypically or suggested by many reads aligning specifically to numerous *E. muscae* transcripts (Webster *et al.* 2015; Shi *et al.* 2016; De Fine Licht *et al.* 2017; Gofton *et al.* 2022). This co-occurrence of fungus and virus appears in a variety of dipteran hosts, including *D. melanogaster*, *M. domestica*, and *D. radicum*.

## Discussion

We believe these data establish that the viruses we have identified in our *E. muscae* in vitro culture, along with three closely related viruses that have been recently reported (Twyford, H39, and Scerri) are variants of an iflavirus that infects *E. muscae* and is transmitted along with *E. muscae* to and from infected dipteran hosts. We visualized the virus infecting *E. muscae in vitro* by electron microscopy (Fig. 3), where its persistent and abundant presence implies propagation, and perhaps active replication, within fungal cells. As the fungal infection proceeds in flies, we observe viral reads rising concordantly with fungal reads at the 72 and 96 h time points (Fig. 2), although there is variation in the fungal:viral ratio between time points and between samples. This variation could be explained by the stochastic dynamics of EV replication within fungal cells and by variability in the immune responses of fungus or fly to the infection, as well as variation in the number of viral particles that become packaged inside of infectious fungal spores. Alternatively, the virus could be transmitted vertically within the fungus in the absence of active replication. Where the virus has been found in nature, it has always been associated with *E. muscae* infection (Fig. 4). For these reasons, we formally propose naming this virus Entomophthovirus (EV), containing the closely related BEV, DrEV, MdEV, Twyford, H39, and Scerri viruses (Fig. 1b).

The consistent association between *E. muscae* and EV suggests that, in spite of an active antiviral response from the fungus (as evidenced by small RNA production), the virus is rarely cleared from fungal cells and continues to be propagated in fungal spores. The extent of virus-associated fungal mortality is unclear.

Another possibility is that EV provides some fitness advantage to *E. muscae* and its presence is essential for successful transmission of the fungal infection in flies. One tantalizing possibility is that the virus is involved in behavior manipulation. Many animal viruses have behavioral effects on their hosts. These include a baculovirus that induces summiting behavior in caterpillars (Katsuma *et al.* 2012) and a virus that manipulates parasitoid wasps towards infecting already-infected larvae so as to increase its opportunities for horizontal transmission (Patot *et al.* 2009).

This catalog of viruses implicated in behavior manipulation includes other Iflaviridae. For example, Kakugo virus, which is a subtype of Deformed Wing Virus, is an iflavirus that infects honeybees and has been shown to be associated with aggressive colony behavior (Fujiyuki *et al.* 2005). Another iflavirus, *Dinocampus coccinellae* paralysis virus (DcPV), infects a parasitoid wasp that manipulates the behavior of ladybeetles. The parasitoid *D. coccinellae* deposits an egg and DcPV in the ladybeetle *Coleomegilla maculata*. The larval *D. coccinellae* exits the ladybeetle, but the virus remains infecting the ladybeetle's nervous system, paralyzing the ladybeetle so that the *D. coccinellae* larvae can develop under the protection of its paralyzed bodyguard (Dheilly *et al.* 2015). Finally, an iflavirus was found associated with *Bombyx mori* infected with the behavioral manipulating ascomycete fungus *Cordyceps militaris* (Suzuki *et al.* 2015), although the nature of the fungal–viral association and its significance are unknown.

While many fungi have been found to harbor RNA viruses (Myers *et al.* 2020), to our knowledge this is the first known member of the viral order Picornavirales to infect a fungus. Most other known Iflaviridae are insect pathogens, suggesting that the virus may have moved from an insect host to an ancestor of *E. muscae*, perhaps during coinfection of fungus and virus in a dipteran host. When this happened and how broad the association is remains to be seen, but there is precedent for host switching in Iflaviridae, most dramatically represented by an iflavirus that has evolved to infect tomato plants, likely also derived from an insect-infecting ancestor (Fig. 1b) (Saqib *et al.* 2015). *Varroa destructor* mites can vector viruses between honeybee colonies, and some of these viruses—Deformed Wing Virus and *Varroa destructor* Virus 1 (a.k.a. Deformed Wing Virus B)—have been shown to actively replicate in the mite as well as the bee, demonstrating further host flexibility to include both insects and chelicerates (Fig. 1b) (Ongus *et al.* 2004; Damayo *et al.* 2023). Other Iflaviridae have been identified in ticks (Daveu *et al.* 2021) and the transcriptomic survey that identified Hubei picorna-like virus 39 also identified novel Iflaviridae in both a spider and a centipede (Shi *et al.* 2016), further increasing the diversity of possible non-insect hosts.

The identification of Scerri virus and its co-occurrence with *E. muscae* in the transcriptomes of Australian ticks has no single clear explanation but instead suggests a few possibilities that remain to be tested. This could represent an additional jump in host specificity for the *E. muscae*/EV complex, which would be unprecedented given that no *Entomophthora* species has been found to infect a non-insect host (Elya and De Fine Licht 2021). Alternatively and consistent with the low read counts of both virus and fungus in those samples, this could represent contamination of the tick by EV-infected *E. muscae*, perhaps through spores landing on the tick surface or acquired through a blood meal. For instance, this strain of *E. muscae*/EV may have evolved to infect a vertebrate-biting dipteran (e.g. mosquito), which might share a feeding source with *H. bancrofti* ticks. Another possibility is that a vector such as a mite could have transferred *E. muscae* and EV from a dipteran to a vertebrate, from which it could be passed on to a biting tick. Mites have been shown to transfer pathogens in both insects (Stone *et al.* 2024) and vertebrates (Herrera-Mares *et al.* 2022). Other non-ecological possibilities for cross-contamination could also explain the data. Further sampling and analysis of Australian *H. bancrofti* ticks will be necessary to understand the exact origin of *E. muscae* and EV reads in these samples.

Many important questions regarding the relationship between EV and *E. muscae* remain open. How widespread is the association between entomopathogenic fungi and viruses? Can *E. muscae* cleared of virus still infect flies and alter behavior? Does the virus replicate in fly cells during infection or is it restricted to the fungus? Can the virus infect flies in the absence of fungus? Does EV facilitate *E. muscae* infection, for instance through immune system modulation (Wang *et al.* 1987; Ives *et al.* 2011; Weinersmith 2019) or even by itself eliciting behavioral changes? Whether EV is involved in behavioral manipulation or not, it is a fascinating example of how a major viral lineage can adapt to a very different host and ecological role. Understanding how this relationship evolved and is maintained promises to illuminate new aspects of virus biology.

## Supplementary Material

jkae198_Supplementary_Data

## Data Availability

The BEV-a and BEV-b genomes (with annotated ORFs) are uploaded to GenBank as PQ096845 (BEV-a) and PQ096846 (BEV-b). Small RNA sequencing is uploaded to the NCBI SRA as part of Project PRJNA1107943, while mRNA sequencing from the *in vitro E. muscae* culture is uploaded to the NCBI SRA as part of Project PRJNA1139198. Supplementary files contain all data necessary to replicate the graphs shown in the figures. The *E. muscae* strain is available upon request. Supplemental material available at G3 online.
